# Beyond the numbers: interpreting the rise in student suicide deaths in India

**DOI:** 10.1016/j.lansea.2026.100822

**Published:** 2026-07-21

**Authors:** Rakhi Dandona, G. Anil Kumar, Rajesh Sagar

**Affiliations:** a PHFI Injury Prevention Research Centre, Public Health Foundation of India, New Delhi, India; b Institute for Health Metrics and Evaluation, University of Washington, Seattle, USA; c Centre for Mental Health and Community Wellbeing, Melbourne School of Population and Global Health, The University of Melbourne, Australia; d All India Institute of Medical Sciences, New Delhi, India

Recent media reports highlighting an increase in student suicide deaths in India between 2023 and 2024 have renewed public concern about mental health among young people.^1^, ^2^, ^3^ According to recent National Crime Records Bureau (NCRB) data, student suicides increased by 4.3% from 13,892 in 2023 to 14,488 in 2024.^4^ At the same time, the overall national suicide rate reportedly declined marginally from 12.3 to 12.2 per 100,000 population.^4^ These trends have been interpreted as evidence of a worsening crisis among students.^1^^,^^2^ The concern is justified, but the interpretation of these data requires caution.

Much of the public discussion has focused on rising numbers, while suicide rates are the more appropriate metric for understanding change over time rather than absolute numbers alone, as highlighted elsewhere.^5^ However, student suicide rates cannot presently be calculated with confidence because NCRB data provide the numerator—the number of deaths classified as students—but not the denominator of all students enrolled across school and higher education systems. Absolute numbers are influenced by population growth, educational expansion, and changing patterns of enrolment.^6^ Without denominator data, it is difficult to determine whether the observed increase reflects rising risk, greater enrolment, improved classification, or a combination of these factors. At the same time, an exclusive emphasis on methodological caution risks obscuring the reality that every increase in the number of deaths represents a preventable loss of life. The challenge is not to choose between numbers and rates, but to recognise the limitations and value of both.

Another concern is how “student” is defined in NCRB statistics. The category originates from professional classification, indicating that the person who died by suicide was a student at the time of death.^4^ Consequently, the label “student suicide” often creates an immediate assumption that the precipitating stressor is academic in nature. Public and media narratives, therefore, frequently focus on examination pressure, competitive educational environments, and institutional stress.^1^, ^2^, ^3^ However, NCRB data do not permit a nuanced understanding of risk factors specifically among students, as these data are not provided by profession category.^4^ Furthermore, “students” are not a homogeneous population. This category will include school-going children and adolescents, as well as those studying in colleges, universities, or other forms of higher education. Extrapolating NCRB data for <18 years age group for the school-going children and adolescents, failure in examination accounted for 9.6% of all suicides in 2024. The major causes were family problems (27.7%), illness (12%), love affairs (14.9%), and other causes (17.7%). Considering NCRB data on the causes of suicide for those aged 18–29 years for those pursuing higher education, failure in examination accounted for 1.6% of all suicides in 2024, with family problems (33.2%), illness (12.8%), and love affairs (8.9%) dominating the list. This indicates that the dominant public narrative may be overly narrow. Academic pressure undoubtedly contributes to distress for many young people in India, but reducing student suicide to “exam stress” risks obscuring the complexity of vulnerability and may inadvertently limit the scope of prevention strategies.

Additionally, because the “students” encompass school-going children to young adults, the social environments, developmental contexts, and support systems for these groups differ considerably. Interventions appropriate for school students may not be sufficient for university students, and vice versa. School-based interventions may include socio-emotional learning, teacher sensitisation, anti-bullying programmes, family engagement, and early identification and referral of distress.^7^^,^^8^ Higher education settings may additionally require institutional mental health services, peer-support programmes, crisis-response systems, mentorship structures, and mechanisms to address discrimination, isolation, financial insecurity, and academic pressure.^9^ Treating all “student suicides” as a single category risks flattening important differences in risk and prevention needs.

India already has an important policy framework in the National Suicide Prevention Strategy, which recognised suicide as a major public health challenge and called for multi-sectoral action.^10^ The Supreme Court of India has also recognised the seriousness of suicides among students and has issued guidance directed primarily at higher education institutions, indicating that their accountability for student well-being extends beyond academic performance as educational spaces function as living and social environments where students experience multiple forms of stress and vulnerability.^11^ However, we still know relatively little about how educational experiences, family adversity, mental health conditions, socioeconomic stress, caste and gender discrimination, digital environments, and substance use interact in shaping suicide risk among students in India.^12^^,^^13^ India therefore needs better linkage between education, health, and social welfare data to understand suicide risk among students. Psychological autopsy studies, longitudinal cohorts, and institution-level reporting could provide more meaningful insights than aggregate annual NCRB counts alone. The recent attention to student suicides should become an opportunity to strengthen evidence-informed prevention, as India still lacks nuanced data on how risk differs across school and higher education settings. Without such evidence, prevention efforts will continue to rely on simplified narratives, that only partially explain a much more complex crisis, with a risk of normalising the continued loss of young and productive lives.

## Contributors

RS conceptualized the idea, RD drafted the manuscript, GAK performed the data analysis with contributions from RD, and RS and GAK provided critical inputs for revision. GAK and RD verified the data underlying this manuscript. All authors approved the final version.

## Declaration of interests

Authors have no competing interests.
